# Comparing Mental Health of School-Age Children with and without Epilepsy

**Published:** 2019

**Authors:** Khodayar OSHVANDI, Marzieh JAHANI SAYAD NOVEYRI, Salam VATADOST

**Affiliations:** 1 Mother & Child Care Research Center, School of Nursing and Midwifery, Hamadan University of Medical Sciences, Hamadan, Iran; 2 Faculty of Nursing and Midwifery, Hamadan University of Medical Sciences, Hamadan, Iran

**Dear Editor-in-Chief**

The article entitled “Comparing Mental Health of School-Age Children with and without Epilepsy” published in summer 2016 (1) was very interesting and focused on a valuable subject, which indicates the researchers’ scrutiny. We would, therefore, like to express our gratitude to the authors for selecting this subject and to the Iranian Journal of Child Neurology as well. 

Given the key role of articles in decisions made for health, correct styles of writing the papers and the accuracy of reviewers when deciding on the final approval can greatly help with scientific advancement. Here presented some points to be taken care of.

Proposal plays a large part in steering the study as a general map (2), and methods are one of the main parts of the paper that should be well addressed (3). As the title of the present article suggests, the researchers should have aimed for conducting a case-control study; they, however, seem to have failed, since the content lacks the characteristics normally observed in case-control studies. As you are well aware, case-control studies tend to compare a healthy group with a group of patients or people with some problems in terms of exposure to some risk factors in the past (4). These studies are mainly conducted to determine the significance of potential relationships between exposure to a factor and developing a disease (5). Moreover, Odds Ratio is used in these types of studies to accurately estimate the relative risk (4-7); nevertheless, the present study does not mention the Odds Ratio and the confidence interval. Given the subject of mental health in children with a history of epilepsy, this study seems to be cross-sectional in type.

Failing to properly explain the samples, using unmatched environments in the case and control groups’ and assigning twice as many subjects to the control group as to the other group without giving a reference constitute the other shortcomings of the paper(8). In addition, the scoring of the data collection tool and its validity and reliability are not addressed in Methods (9). Key terms should be defined in objectives (10), introduction is recommended to be enriched by presenting terms and definitions. Moreover, asking subjects to sign informed consents as the only ethical observation mentioned by the author should be complemented by mentioning the Ethics Committee approval code and the method of receiving the informed consent (10). Similarly, acknowledgments only encompass the sponsor and failed to include the participants. The majority of references date back to before 2010 and only six references are associated with 2010-2016; more recent publications are therefore recommended to be added to references. 

In the end, we would like to congratulate the researcher and the colleagues for accurately addressing such an interesting topic and we hope they always pioneer this field by observing these minor points as cited.
